# Wunden durch Vaskulitiden – aktuelle Klassifikation, Diagnostik und Therapie

**DOI:** 10.1007/s00391-023-02166-3

**Published:** 2023-03-09

**Authors:** Joachim Dissemond

**Affiliations:** Klinik und Poliklinik für Dermatologie, Venerologie und Allergologie, Universitätsklinikum Essen, Hufelandstr. 55, 45122 Essen, Deutschland

**Keywords:** Chapel-Hill-Konsensus Konferenz, Purpura, Inflammation, Wunden, Schmerzen, Chapel Hill consensus conference, Purpura, Inflammation, Wounds, Pain

## Abstract

Wunden an der Haut können sehr unterschiedliche Ursachen haben. Insbesondere bei klinisch atypischen oder nicht-heilenden Wunden ist die sehr heterogene Gruppe der Vaskulitiden von besonders wichtiger differenzialdiagnostischer Bedeutung. Die Klassifikation der Vaskulitiden erfolgt heute entsprechend den betroffenen Gefäßen nach der Chapel-Hill-Konsensus-Konferenz. Von einer Vaskulitis kann potenziell jeder Teil des Gefäßsystems betroffen sein. Dadurch wird deutlich, dass oft die Gefahr von systemischen Erkrankungen mit hoher interdisziplinärer Relevanz besteht.

Klinisch entwickeln sich die in der Regel sehr schmerzhaften Wunden bei kutaner Vaskulitis aus Nekrosen und sind typischerweise in der floriden Phase von einem erythematös-lividen Randsaum umgeben. In der meist umfangreichen Diagnostik hat zusätzlich zu der klinischen Inspektion die histopathologische Untersuchung von Biopsien einen besonders großen Stellenwert.

Therapeutisch sollte immer eine adäquate Wundtherapie mit dem Fokus auf Schmerzvermeidung und Infektionsprophylaxe durchgeführt werden. Bei begleitenden Ödemen unterstützt die Kompressionstherapie zudem die Wundheilung. Darüber hinaus ist es oft notwendig, systemische Therapien mit immunsuppressiven oder immunmodulierenden Medikamenten einzuleiten. Wann immer möglich, sollten die ursächlich relevanten Faktoren und Komorbiditäten frühzeitig diagnostiziert und vermieden bzw. behandelt werden. Andernfalls besteht die Gefahr von schweren oder sogar tödlichen Krankheitsverläufen.

## Abstract (en)

Wounds of the skin can have very different causes. Especially in clinically atypical or non-healing wounds, the very heterogeneous group of vasculitides is of particularly important differential diagnostic significance. Nowadays, the classification of vasculitis is based on the affected vessels according to the Chapel Hill consensus conference. Thus, potentially any part of the vascular system can be affected. It becomes clear that there is often a risk of systemic diseases with high interdisciplinary relevance.

Clinically, the usually very painful wounds in cutaneous vasculitis develop from necrosis and are typically surrounded by an erythematous-livid rim in the florid phase. In addition to clinical inspection, the histopathological examination of biopsies is of particular importance in the usually extensive diagnostic work-up.

Therapeutically, adequate wound treatment should always be performed with a focus on pain prevention and infection prophylaxis. In the case of edema, compression therapy also supports wound healing. In addition, it is often necessary to initiate systemic treatment with immunosuppressive or immunomodulating drugs. Whenever possible, causally relevant factors and comorbidities should be diagnosed early and avoided or treated. Otherwise, there is a risk of severe or even fatal disease progression.

Vaskulitiden sind eine heterogene Gruppe von Erkrankungen, bei denen es durch autoimmunologische Prozesse zu Entzündungen in den Gefäßwänden kommt. In der Folge kann es zu Stenosen, Okklusionen und/oder Aktivierung des Gerinnungssystems mit Thrombosierung kommen. Da grundsätzlich alle Gefäße im menschlichen Körper von einer Vaskulitis betroffen sein können, werden die Patienten mit sehr unterschiedlichen Symptomen in vielen verschiedenen medizinischen Fachbereichen behandelt. Oft handelt es sich somit um multidisziplinär relevante Krankheitsbilder.

## Klassifikation

Die Prävalenz der Vaskulitiden liegt bei ca. 200 pro Mio. Einwohner; die Inzidenz beträgt pro Jahr etwa 50 pro Mio. Einwohner. Das Risiko, an einer Vaskulitis zu erkranken, steigt mit zunehmenden Lebensalter deutlich an [28]. Die Klassifikation der Vaskulitiden erfolgt meist entsprechend der Chapel-Hill-Konsensus-Konferenzen (CHCC). Diese wurden erstmalig 1994 und dann 2012 durchgeführt und klassifiziert die Krankheitsbilder in erster Linie entsprechend der betroffenen Gefäßtypen und -kaliber ([14]; Tab. 1). Eine exakte Zuordnung der Vaskulitiden ist für die weiterführende individuelle Diagnostik, Prognose und therapeutische Intervention von großer Bedeutung. Da der zentral wichtige Aspekt bei der Zuordnung die Diagnostik der betroffenen Gefäße ist, wird deutlich, dass eine histopathologische Untersuchung von Biopsien erforderlich ist. Die in der CHCC als klein bezeichneten Gefäße liegen in der Dermis, die mittelgroßen Gefäße in der Subkutis, und die großen Gefäße sind die Aorta sowie die hier abgehenden Äste. Bei den meisten Vaskulitiden handelt es sich um systemische Erkrankungen, die potenziell mehrere Organsysteme betreffen können. Darüber hinaus werden aber auch Vaskulitiden einzelner Organe wie beispielsweise der Haut beschrieben.

**Table 1:** 

Vaskulitis kleinerer Gefäße	*ANCA-assoziierte Vaskulitis*
	Eosinophilie Granulomatose mit Polyangiitis (Churg-Strauss-Syndrom)
	Granulomatosis mit Polyangiitis (Wegener)
	Mikroskopische Polyangiitis
	*Immunkomplexvaskulitis*
	Antikörpervermittelte Krankheit der glomerulären Basalmembran (Anti-GBM-Erkrankung)
	Kryoglobulinämische Vaskulitis
	Hypokomplementämische Vaskulitis (Anti-C1q-Vaskulitis)
	IgA-Vaskulitis (Henoch-Schoenlein-Purpura)
Vaskulitis mittelgroßer Gefäße	*Polyarteriitis nodosa*
	*Kawasaki-Syndrom*
Vaskulitis größerer Gefäße	*Takayasu-Arteriitis*
	*Riesenzellarteriitis*
Vaskulitis variabler Gefäßgrößen	*Morbus Behçet*
	*Cogan-Syndrom*
Vaskulitis einzelner Organe	*Kutane leukozytoklastische Angiitis (*^a^*kutane IgM/IgG-Immunkomplex-Vaskulitis)*
	*Primäre ZNS-Vaskulitis*
	*Isolierte Aortitis*
	*Andere*^a^
	Noduläre kutane Vaskulitis (Erythema induratum Bazin)
	Erythema elevatum et diutinum
	Rezidivierende makuläre Vaskulitis bei Hypergammaglobulinämie
	Normokomplementämische urtikarielle Vaskulitis
Mit systemischer Erkrankung assoziierte Vaskulitis/systemische Vaskulitis	*Lupus-Vaskulitis*
	*Rheumatoide Vaskulitis*
	*Sarkoidose-Vaskulitis*
	*Andere*
Mit wahrscheinlicher Ätiologie assoziierte Vaskulitis/sekundäre Vaskulitis	*Malignomassoziierte Vaskulitis*
	*Medikamentenassoziierte ANCA-assoziierte Vaskulitis*
	*Medikamentenassoziierte Immunkomplexvaskulitis*
	*Hepatitis-B-Virus-assoziierte Vaskulitis*
	*Hepatitis-C-Virus-assoziierte kryoglobulinämische Vaskulitis*
	*Syphilis-assoziierte Aortitis*
	*Andere*

^a^Einschließlich des dermatologischen Addendums [24]

## Diagnostik

Die strukturierte Diagnostik bei Wunden an der Haut kann entsprechend der ABCDE-Regel durchgeführt werden. Hierbei steht A für Anamnese, B für Bakterien, C für klinische Untersuchung, D für Durchblutung und E für Extras wie beispielsweise Biopsien [2]. Die Verdachtsdiagnose einer Vaskulitis kann meist schon nach Anamnese und klinischer Untersuchung der typischen Hautveränderungen gestellt werden. Für die Sicherung der Diagnose ist es aber notwendig, so frühzeitig wie möglich Biopsien aus den Hautveränderungen zu entnehmen. Falls bereits Wunden vorliegen, sollte eine Spindelbiopsie aus dem floriden Wundrandbereich durchgeführt werden, die bis in die Subkutis reicht. Neben der konventionellen histopathologischen Untersuchung mit Hämatoxylin-Eosin(HE)-Färbung, sollte auch eine direkte Immunfluoreszenz (DIF) veranlasst werden, um eine exakte Zuordnung der Vaskulitis zu ermöglichen [9]. Zu beachten ist, dass die in den histopathologischen Befunden beschriebenen entzündlichen Infiltrate der Gefäßwände nicht spezifisch für eine primäre Vaskulitis sind, sondern oft auch bei anderen Wunden wie beispielsweise bei Ulcus cruris venosum als sekundäres, unspezifisches Phänomen in der Wundumgebung gefunden werden können [9]. Für die individuelle Beurteilung der Patienten müssen dann sowohl die histopathologischen Befunde als auch die Anamnese, serologische Ergebnisse und der klinische Befund gemeinsam berücksichtigt werden.

Bei der Basisdiagnostik der Patienten mit Vaskulitis sollten zumindest Blutbild, CRP, BSG, Urin sowie Leber- und Nierenwerte bestimmt werden. Beispielsweise Autoantikörper, Blutgerinnung, Differenzialblutbild, Komplementfaktoren, Serumproteinelektrophorese, Kryoglobuline, peripherer Blutausstrich, Streptokokkenantikörper und Virentests sollten erst bei den weiterführenden Untersuchungen abgenommen werden. Bevor diese weitere serologische und ggf. eine bildgebende Diagnostik veranlasst wird, ist es oft sinnvoll, zuerst die histopathologischen Befunde abzuwarten, um diese Untersuchungen dann gezielt individuell zu planen [11].

## Klinische Befunde

Bei vielen Patienten mit einer Vaskulitis kommt es zu Hautveränderungen, die bei den verschiedenen Krankheitsbildern sehr unterschiedlich aussehen können. Vaskulitische Hautveränderungen beginnen oft mit Juckreiz und/oder Schmerzen. In Abhängigkeit von einer Organbeteiligung können zudem weitere Symptome wie beispielsweise Abgeschlagenheit, Fieber, Arthritis, gastrointestinale Beschwerden, Proteinurie oder Sehstörungen auftreten [8].

**Figure 1:**
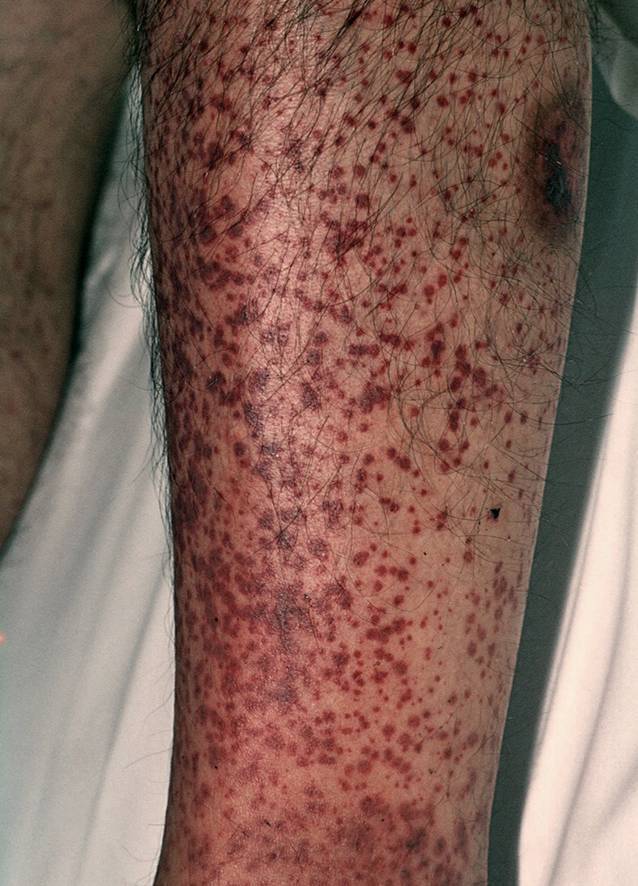

**Figure 2:**
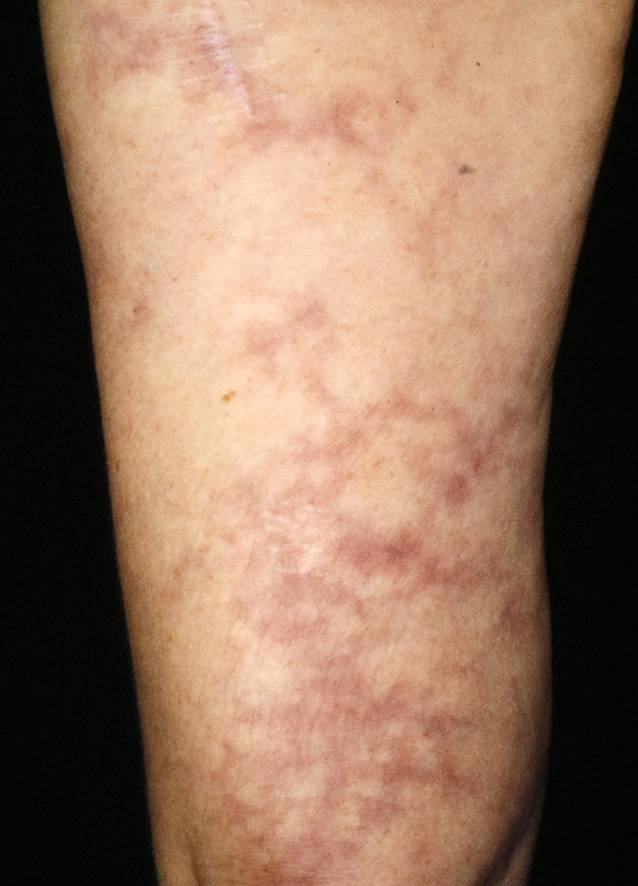

Wenn die kleinen Gefäße von einer Vaskulitis betroffen sind, kommt es zu Erythrozytenextravasion, Ödemen und Hämorrhagien. Klinisch resultiert an der Haut meist eine palpable Purpura, ggf. auch hämorrhagische Blasen (Abb. 1). Die Hautveränderungen beginnen oft an den unteren Extremitäten und breiten sich von distal nach proximal aus. Sind mittelgroße Gefäße betroffen, kommt es eher zu subkutanen, druckschmerzhaften Knoten an der Haut. Beispielsweise kommt es bei der Polyarteriitis nodosa durch thrombotische Verschlüssen der Hautgefäße zu einer typischen, aber nicht spezifischen Livedo racemosa (Abb. 2). Bei den Vaskulitiden der größeren Gefäße ist eine Beteiligung der Haut eher selten. Es kommt eher zu Zephalgie, Subclavian-Steal-Syndrom oder Claudicatio.

## Differenzialdiagnosen

**Figure 3:**
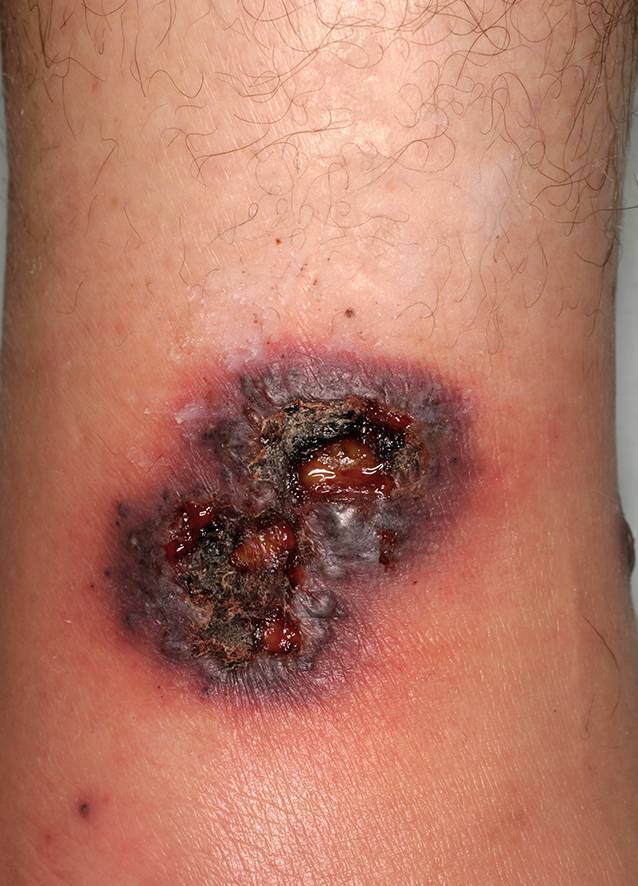

Patienten mit Wunden werden in vielen verschiedenen Fachbereichen der Medizin behandelt. Diese Wunden werden in Deutschland als chronisch bezeichnet, wenn sie nach acht Wochen nicht abgeheilt sind [4]. In Europa sind die häufigsten chronischen Wunden das diabetische Fußulkus, Ulcus cruris venosum und Dekubitus. Seltenere Ursachen sind inflammatorische Krankheitsbilder, zu denen auch die Vaskulitiden zählen (Abb. 3). Bei der Inflammation kann es klinisch, ebenso wie bei Infektionskrankheiten, zu den typischen klassischen Entzündungszeichen Rubor, Tumor, Calor, Dolor und Functio laesa kommen [21]. Daher sollte insbesondere bei inflammatorischen Wunden unbedingt eine bakteriell verursachte Wundinfektion abgegrenzt werden. Da sich die diagnostische Klärung oft schwierig gestaltet und die Weiterentwicklung in eine Sepsis gefürchtet wird, erhalten viele Patienten ohne Nachweis einer bakteriellen Infektionskrankheit eine nicht notwendige, systemische Antibiotikatherapie. Dieses Vorgehen steht im Widerspruch zu den heute propagierten Antibiotic-Stewardship(ABS)-Bemühungen [25]. Eine praktische Hilfe im klinischen Alltag für die Diagnostik lokaler Wundinfektionen kann der validierte Score Therapeutischer Index für lokale Infektionen (TILI) sein [6]. Zudem müssen die entzündlichen Vaskulitiden auch von den primär nicht-entzündlichen Vaskulopathien mit partieller oder totaler Okklusion des Gefäßlumina abgegrenzt werden [22]. Als gemeinsame Endstrecke kann es klinisch zu sehr ähnlich aussehenden Wunden kommen. So ist beispielsweise die klassische klinische Trias der Livedovaskulopathie die Livedo racemosa, Atrophie blanche und sehr schmerzhafte Ulzera. Auch die sehr schmerzhaften Ulzerationen bei Pyoderma gangraenosum können mit einer Vaskulitis verwechselt werden. Hierbei handelt es sich aber um eine seltene, primär sterile, neutrophile Dermatose, die im Rahmen eines Pathergie-Phänomens oft nach einem (Minimal‑)Trauma mit den Prädilektionsstellen an den Streckseiten der unteren Extremitäten auftritt. Für die Diagnostik des Pyoderma gangraenosums kann heute der validierte PARACELSUS-Score genutzt werden [15].

Da die notwendigen Therapieansätze bei den verschiedenen Krankheitsbildern unterschiedlich sind, müssen diese Differenzialdiagnosen unbedingt korrekt diagnostiziert werden (Infobox 1).

## Wunden und Vaskulitis

Grundsätzlich können alle Vaskulitiden, die sich an der Haut manifestieren, Wunden verursachen. Die transmurale Entzündung kann zu einer vollständigen Destruktion der Gefäße führen. Es resultiert dann eine Minderperfusion in den zu versorgenden Hautarealen, was zu einer fokalen Ischämie, Nekrosen und schließlich zu Ulzerationen führt [20]. Ob eine Vaskulitis bei den Patienten tatsächlich eine Wunde verursacht, ist von verschieden Faktoren abhängig. Relevant sind hierbei u. a. das Kaliber der betroffenen Gefäße, das Vorliegen einer Hyperkoagulabilität, die anatomische Lokalisation und begünstigende Kofaktoren, wie beispielsweise Diabetes mellitus oder vaskuläre Erkrankungen [17, 23]. Am häufigsten entstehen Wunden an der Haut durch eine kutane leukozytoklastische Angiitis. Akute Auslöser für diese Krankheitsaktivität können beispielsweise Medikamente und/oder Infektionskrankheiten sein. Allerdings müssen auch zugrunde liegende Systemerkrankung und Neoplasien beachtet werden (Infobox 2).

**Figure 4:**
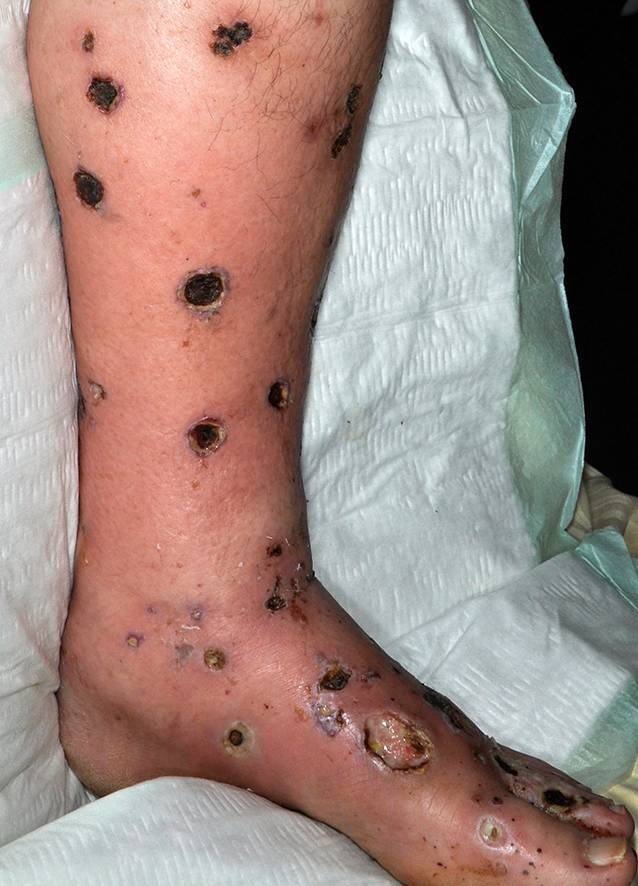

Wunden durch Vaskulitis können grundsätzlich an allen Haut- und Schleimhautarealen entstehen. Am häufigsten findet man diese jedoch an den distalen unteren Extremitäten. Dort kommt es oft beidseits zu multiplen Ulzerationen (Abb. 4; [20]). Typisch für diese Wunden ist, dass sie sich rasch vergrößern, livide Ränder aufweisen, eher scharf begrenzt und sehr schmerzhaft sind. Oft sind mehrere anatomische Areale betroffen. Begleitend treten insbesondere an den unteren Extremitäten oft Ödeme auf. Wenn die Inflammation rückläufig ist, kommt es zu postinflammatorischen Hyperpigmentierungen an der Haut. Die Abheilung der Ulzerationen erfolgt obligat mit Narben [13].

## Therapie

Das zentral wichtige Ziel der Therapie der Patienten mit Wunden durch Vaskulitiden ist es, die Entzündungsreaktion möglichst frühzeitig und suffizient zu unterdrücken, um Folgekomplikationen zu verhindern [20]. Wenn relevante Grundkrankheiten bzw. Auslöser diagnostiziert werden, sollte deren Behandlung bzw. Meidung der erste Schritt in der komplexen Behandlungsstrategie sein.

Es gibt nur sehr wenige evidenzbasierte Empfehlungen für die Behandlung von Patienten mit Wunden durch Vaskulitiden. Die Auswahl der Therapie orientiert sich individuell an der Ausprägung und den Manifestationsorten der Vaskulitis. Auch wenn der Stellenwert der topischen Therapie mit hochpotenten Glukokortikoiden in der Wundumgebung bislang wissenschaftlich nicht eindeutig belegt werden konnte, ist deren Anwendung heute meist ein wichtiger Bestandteil der Therapiekonzepte [1]. Alternativ kann insbesondere für den längerfristigen Einsatz eine Tacrolimus-Salbe im off-label use eingesetzt werden [29].

Eine systemische Behandlung sollte frühzeitig bei schwerwiegenderen Verläufen zum Einsatz kommen. Meist sind Glukokortikoide weiterhin die Mittel der ersten Wahl. Weitere immunsuppressive Alternativen für die systemische Therapie sind beispielsweise Azathioprin, Ciclosporin, Cyclophosphamid, Hydroxychloroquin, MTX und Mycophenolat-Mofetil oder immunmodulierende intravenöse Immunglobuline [5, 20]. Insbesondere bei therapierefraktären Verläufen und/oder ANCA-assoziierten Vaskulitiden werden heute auch monoklonale Anti-CD20-Antikörper wie Rituximab erfolgreich eingesetzt [26]. Neue therapeutische Ansätze sind zudem weitere Biologika, die beispielsweise TNF‑α, IL‑1, -5, -6, -17, -12 und -23 Antikörper oder sog. Small Molecules wie JAK-Inhibitoren enthalten [18, 27].

## Besondere Aspekte des Wundmanagements

Bei der Wundbehandlung von Patienten mit Vaskulitis sollten grundsätzlich die Prinzipien der modernen, an den Phasen der Wundheilung orientierte, feuchte Wundversorgung beachtet werden [3]. Da eine Besonderheit die ausgeprägte Schmerzhaftigkeit ist, sollte auf atraumatische Verbandwechsel und ggf. auch die Implementierung einer Schmerztherapie geachtet. Hier können lokal wirksame Schmerzmittel wie beispielsweise Ibuprofen [10] in Wundauflagen oder Morphium in einem Wundgel [12] eingesetzt werden (Tab. 2). Bei den meisten Patienten ist allerdings zusätzlich eine begleitende systemische analgetische Therapie entsprechend dem WHO-Stufenschema notwendig. Da viele Patienten eine systemische immunsuppressive Therapie erhalten, sollten auch auf eine antimikrobielle Wundtherapien geachtet werden. Hier werden meist Wundprodukte empfohlen, die Polihexanid (PHMB), Octenidin oder Silber beispielsweise in Form von Hydrogelen oder Wundauflagen enthalten [16].

**Table 2:** 

Morphinhydrochloridtrihydrat	0,1 g
Natriumedetat	0,1 g
Hydroxyethylzellulose 250 G	4,5 g
Polihexanidkonzentrat 20 %	0,2 g
Aqua dest.	ad 100 g

Wenn die inflammatorische Aktivität der Vaskulitis gut kontrolliert ist, können im weiteren Verlauf der Behandlung nahezu alle Verfahren der Wundbehandlung, inklusive Vakuumtherapie oder Spalthauttransplantation, eingesetzt werden [19]. In frühen inflammatorischen Phasen sind diese physikalischen Therapien allerdings meist kontraindiziert [20].

Da die Kompressionstherapie u. a. Ödeme reduziert und den venösen Rückstrom beschleunigt, profitieren die meisten Patienten mit Wunden durch Vaskulitis an den unteren Extremitäten auch von einer begleitenden Kompressionstherapie [8]. Die Patienten tolerieren initial oft aber keine hohen Ruhedruckruckwerte, sodass auch Kompressionssysteme mit niedrigen Ruhedruckwerte um 20 mmHg für die Therapie eingesetzt werden können [22]. Dies kann im klinischen Alltag meist gut mit „Lite“-Varianten der Mehrkomponentenverbände, medizinischen adaptiven Kompressionsbandagen (MAK), bei denen der Druck über Klettverschlüsse eingestellt wird oder medizinische Kompressionsstrümpfe der Klasse 1 umgesetzt werden [7].

### Infobox 1 Auswahl von klinischen Differenzialdiagnosen bei Wunden durch Vaskulitiden

- Bullöse Autoimmunerkrankungen, z. B. bullöses Pemphigoid, Epidermolysis bullosa acquisita
- Gerinnungsstörungen, z. B. Antiphospholipidsyndrom, Faktor-V-Leiden-Mutation
- Hämatologische Erkrankungen, z. B. Leukämie, Thalassämie
- Kalziphylaxie
- Necrobiosis lipoidica
- Pannikulitiden
- Porphyria cutanea tarda
- Pyoderma gangraenosum
- Ulcus cruris hypertonicum Martorell
- Vaskulopathie, z. B. Livedovaskulopathie

### Infobox 2 Auswahl von Ursachen für eine kutane leukozytoklastische Angiitis

- Medikamente, z. B. Allopurinol, Antibiotika
- Infektionskrankheiten, z. B. durch Streptokokken, Hepatitis-C-Virus
- Inflammatorische Autoimmunerkrankungen, z. B. rheumatoide Arthritis, Colitis ulcerosa
- Neoplasien, z. B. Non-Hodgkin-Lymphom, Mammakarzinom

## Fazit für die Praxis

- Wunden an der Haut sind ein Symptom verschiedenster zugrundeliegender Ursachen.
- Wichtige Differenzialdiagnosen, insbesondere von klinisch atypischen Wunden, können Vaskulitiden sein.
- Bei Verdacht auf Vaskulitiden sollte die Diagnose möglichst histopathologisch gesichert werden.
- Meist ist bei Wunden durch Vaskulitiden eine immunsupprimierende oder immunmodulierende systemische Therapie sinnvoll.
- Für die konservative Therapie der Wunden durch Vaskulitis gelten die Grundprinzipien der modernen feuchten Wundtherapie.
